# The optical response of artificially twisted MoS$_2$ bilayers

**DOI:** 10.1038/s41598-021-95700-5

**Published:** 2021-08-23

**Authors:** M. Grzeszczyk, J. Szpakowski, A. O. Slobodeniuk, T. Kazimierczuk, M. Bhatnagar, T. Taniguchi, K. Watanabe, P. Kossacki, M. Potemski, A. Babiński, M. R. Molas

**Affiliations:** 1 Institute of Experimental Physics, Faculty of Physics, University of Warsaw, ul. Pasteura 5, 02-093 Warsaw, Poland; 2 Department of Condensed Matter Physics, Faculty of Mathematics and Physics, Charles University, Ke Karlovu 5, 121 16 Prague 2, Czech Republic; 3 International Center for Materials Nanoarchitectonics, National Institute for Materials Science, 1-1 Namiki, Tsukuba, 305-0044 Japan; 4 Research Center for Functional Materials, National Institute for Materials Science, 1-1 Namiki, Tsukuba, 305-0044 Japan; 5 Laboratoire National des Champs Magnétiques Intenses, CNRS-UGA-UPS-INSA-EMFL, 25, Avenue des Martyrs, 38042 Grenoble, France

**Keywords:** Electronic properties and materials, Two-dimensional materials

## Abstract

Two-dimensional layered materials offer the possibility to create artificial vertically stacked structures possessing an additional degree of freedom—*the* *interlayer* *twist*. We present a comprehensive optical study of artificially stacked bilayers (BLs) MoS$_2$ encapsulated in hexagonal BN with interlayer twist angle ranging from 0$^{\circ }$ to 60$^{\circ }$ using Raman scattering and photoluminescence spectroscopies. It is found that the strength of the interlayer coupling in the studied BLs can be estimated using the energy dependence of indirect emission versus the A$_\text {1g}$–E$_\text {2g}^1$ energy separation. Due to the hybridization of electronic states in the valence band, the emission line related to the interlayer exciton is apparent in both the natural (2H) and artificial (62$^\circ$) MoS$_2$ BLs, while it is absent in the structures with other twist angles. The interlayer coupling energy is estimated to be of about 50 meV. The effect of temperature on energies and intensities of the direct and indirect emission lines in MoS$_2$ BLs is also quantified.

## Introduction

Two-dimensional (2D) van der Waals (vdW) crystals have emerged as a new generation of materials with extraordinary properties. For instance, widely studied semiconducting transition metal dichalcogenides (S-TMDs) transform from indirect- to direct-band gap, optically-bright semiconductors when thinned down to a monolayer (ML), which results in unique electronic structures and consequent optical properties ^1–4^. The family of 2D layered materials grows day by day, hugely expanding the scope of possible phenomena to be explored in two dimensions. Growing is also the number of possible vdW heterostructures that one can create. Such 2D materials currently cover a vast range of properties allowing potential applications in, i.a. spintronic devices ^5^, optoelectronics ^6,7^, tunnel field-effect transistors ^8,9^, single-photon sources ^10,11^, and quantum information processing ^12,13^. Rapid advances in fabrication methods, like chemical vapor deposition (CVD) growth and mechanical exfoliation techniques, have contributed to increased interest in artificial stacking of different layered materials on top of each other. The simplistic approach of producing vertical vdW heterostructures without the constraints of crystal lattice mismatch enables integrating various 2D materials to create diverse systems with new electronic properties that are not present in pristine components. In addition to the selection of compounds in terms of their properties, a new degree of freedom has emerged: *the* *twist* *angle* between stacked layers, which gives rise to the group of the so-called *twistronic* *materials* ^14,15^. The twist angle is responsible for the occurrence of moiré patterns, that leads to new and intriguing phenomena, like the formation of secondary Dirac points in graphene on hexagonal boron nitride (hBN) ^16,17^ or hybridized (moiré) excitons in vdW heterostructures formed by stacked two S-TMD MLs ^18–23^.

Raman scattering (RS) and photoluminescence (PL) spectroscopies are extensively used experimental techniques to characterize layered materials. Particularly, they can be used to determine the thickness of each S-TMD thin layer due to the energy dependence of phonon vibrations ^24–31^ as well as to the direct-indirect band-gap transformation ^1–4^.

In this work, we investigate interlayer interactions in high-quality artificially stacked twisted MoS$_2$ bilayers (BLs) encapsulated in hBN flakes using the RS and PL spectroscopies. Our results indicate that the interlayer coupling can be determined by the comparison of the emission energies of the indirect transition versus the energy separation between two basic intralayer phonon modes (A$_\text {1g}$ and E$_\text {2g}^1$). The origin of the apparent emission due to the interlayer excitons in both the natural (2H) and artificial (62$^\circ$) MoS$_2$ BLs and its absence for the BLs with other twist angles is associated with hybridization of electronic states in the valence band. We also investigate the evolution of energies and intensities of the emission lines due to direct and indirect transitions with temperature ranging from 5 to 300 K.

## Results

### Atomic structures of twisted bilayers MoS$_2$

**Figure 1:**
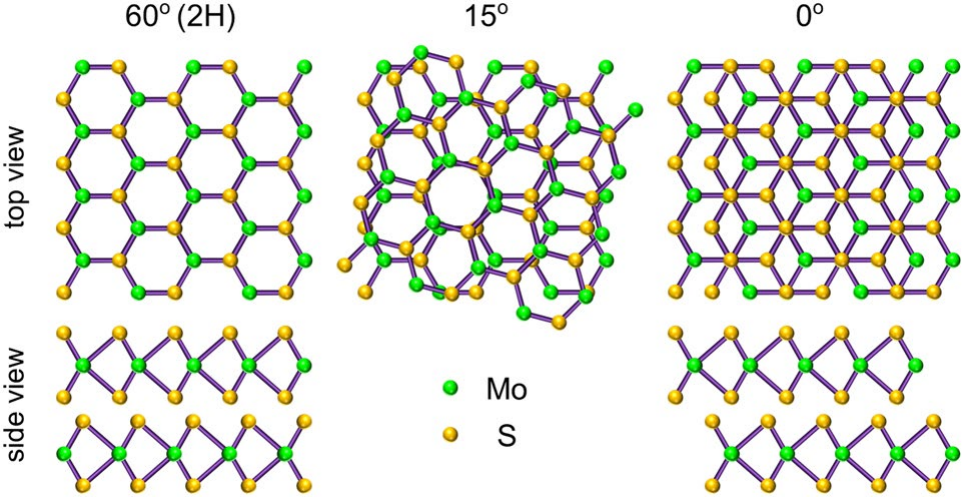
Atomic structure and stacking order of bilayers MoS$_2$ for different twist angles: 60$^\circ$, 15$^\circ$ and 0$^\circ$. Note that the side view demonstrates the energetically favourable structures for angles of 60$^\circ$ and 0$^\circ$.

MoS$_2$ belongs to the family of S-TMDs with chemical formula MX$_2$ where M = Mo or W and X = S, Se or Te, which the most common crystallographic structure is a hexagonal phase. In that case, X–Mo–X atoms in a monolayer (1 L) of MoX$_2$ are arranged in a trigonal prismatic structure, which does not exhibit inversion symmetry. A BL formed by the stacking of two MLs exhibits an additional degree of freedom—the twist angle between the layers. Due to the hexagonal symmetry of a MX$_2$ ML, the different arrangements are characterized by twist angles ranging from 0$^\circ$ to 60$^\circ$. The schematic illustration of three patterns of MoS$_2$ BLs with the twist angles of 60$^\circ$, 15$^\circ$, and 0$^\circ$ are presented in Fig. 1. The most stable structures, which exhibit the strongest coupling, are found for twist angles equal to 0$^\circ$ and 60$^\circ$ ascribed correspondingly to the 3R and 2H stackings^32,33^. We do not use the label 3R for the 0$^\circ$ BL (see Fig. 1), because the 3R unit cell involves the atoms from three consecutive layers (2H unit cell is composed of two layers). While both the 2H and 3R polytypes are accessible in natural and CVD-grown MoS$_2$ multilayers ^33^, the BLs with other twist angles can only be constructed by the artificial stacking of individual monolayers. It is important to point out that the inversion symmetry is restored in the 2H BL, while the zero-twist angle BL has neither inversion nor in-plane mirror.

### Raman scattering spectroscopy

**Figure 2:**
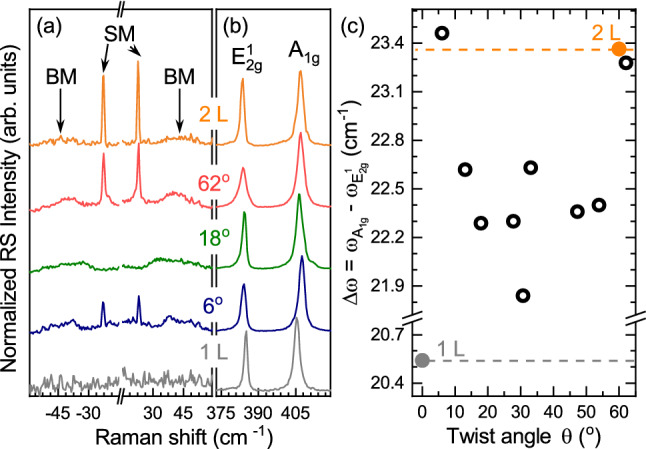
Normalized room-temperature Raman scattering spectra of thin layers MoS$_2$: monolayer (1 L), bilayer (2 L) and selected homobilayers with twist angles of 6$^\circ$, 18$^\circ$ and 62$^\circ$ in the energy range of (**a**) interlayer and (**b**) intralayer vibrations measured under excitation of 2.41 eV laser light. The spectra are normalized to the intensity of the A$_\text {1g}$ peak. (**c**) The energy separation between the A$_\text {1g}$ and E$_\text {2g}^1$ peaks, i.e. $\Delta \omega =\omega _{\text {A}_\text {1g}}-\omega _{\text {E}_\text {2g}^1}$, as a function of twist angle ($\theta$) for all the studied twisted homobilayers. The corresponding $\Delta \omega$ for 1 L and 2 Ls, represented by solid symbols, are also shown.

In order to investigate the interlayer coupling in the twisted BLs and therefore to distinguish their pristine properties, we compare their RS and PL spectra with those obtained for 1 L and 2 L exfoliated from 2H bulk MoS$_2$. Note that the twist angle between MLs forming BLs was determined using the second-harmonic generation (SHG) technique, which permits to reveal the crystallographic orientation of individual MLs^34^. The measured RS spectra of the selected BLs with twist angles of 62$^\circ$, 18$^\circ$, and 6$^\circ$ accompanied with the ones of 1 L and 2 L MoS$_2$ are presented in Fig. 2a, b.

First, we focus on the low-energy range of the RS spectra, presented in Fig. 2. There are no modes related to rigid vibrations in 1 L affirming their interlayer nature. For 2 L, two modes are observed at $\sim$ 23 cm$^{-1}$ and $\sim$ 44 cm$^{-1}$, which are associated with the in-plane shear (SM) and out-of-plane breathing (BM) vibrations of rigid layers, respectively ^33,35,36^. In twisted BLs with angles of 62$^\circ$ and 6$^\circ$, the SM and BM modes are also apparent. This indicates that the coupling between adjacent layers in these two BLs is relatively strong due to their resemblance to the natural 2H and 3R stackings. Note that the relatively smaller intensity of the SM peak in the 6$^\circ$ BLs as compared with the 2H and 62$^\circ$ ones can be attributed to the smaller interlayer bond polarizability for the 3R polytype ^37^. This effect was applied to identify the stacking order of S-TMD layers ^30,37^. On the other hand, only the BM-related peak can be observed for the sample with the 18$^\circ$ angle as well as for other BLs with angles in the range from $\sim$ 10$^\circ$ to $\sim$56$^\circ$ (data not shown). The higher energy range of all RS spectra shown in Fig. 2b is dominated by two phonon modes ascribed to the in-plane ($\mathrm {E^1_{2g}}$) and out-of-plane ($\mathrm {A_{1g}}$) intralayer vibrations. In few layers 2H MoS$_2$, the $\mathrm {E^1_{2g}}$ ($\mathrm {A_{1g}}$) phonon mode experiences a red (blue) shift with increasing the layer thickness. The energy separation between those modes, i.e. $\Delta \omega =\omega _{\text {A}_\text {1g}}-\omega _{\text {E}_\text {2g}^1}$, is a useful tool to determine the number of MoS$_2$ layers ^24^. As can be seen in the Figure, the $\Delta \omega$ energy difference varies for different twist angles ($\Theta$), which is summarized for all samples in Fig. 2c. The $\Delta \omega$ values are largest for samples with a twist angle of 6$^\circ$ and 62$^\circ$ which correspond well to natural 2H and 3R BLs ^38^. In the intermediate cases (6$^\circ< \Theta < 60 ^\circ$), the $\Delta \omega$ falls lower than for 2 L MoS$_2$, but it is considerably larger than in 1 L. The $\Delta \omega$ can be also used to characterize the effective interlayer mechanical coupling strength or, in other words, the distance between layers in twisted BLs ^32^. In such case, we can conclude that the structures with the twist angle equal to 6$^\circ$ and 62$^\circ$ are characterized by the strongest coupling (the smallest interlayer distance), while the coupling strength is weaker (the interlayer distance is bigger) for other BLs with the twist angles substantially different from 0 and 60 degrees.

Note that our RS results are consistent with previous works devoted to the twisted MoS$_2$ BLs exfoliated on Si/SiO$_2$ substrates ^32,39–42^.

### Photoluminescence experiment

**Figure 3:**
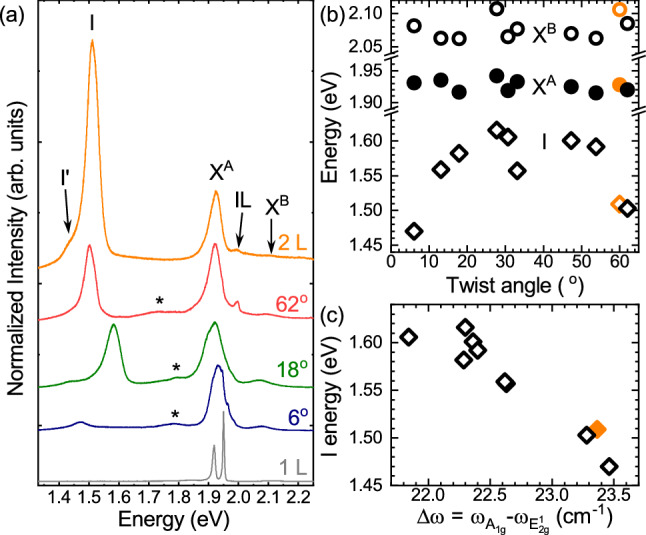
(**a**) Normalized low-temperature ($T=$ 5 K) photoluminescence spectra of thin MoS$_2$ layers: monolayer (1 L), bilayer (2 L) and selected homobilayers with twist angles of 6$^\circ$, 18$^\circ$ and 62$^\circ$ measured under excitation of 2.41 eV laser light. The spectra are normalized to the intensity of the X$^\text {A}$ emission. (**b**) The emission energy of the direct (X$^\text {A}$ and X$^\text {B}$) and indirect (I) transitions as a function of twist angle ($\theta$) for all the studied twisted homobilayers. The full and open black circles represent correspondingly the X$^\text {A}$ and X$^\text {B}$, while the I emissions are indicated by the open black diamonds. (**c**) The evolution of the indirect emission energy, i.e. I, versus the energy separation between the A$_\text {1g}$ and E$_\text {2g}^1$ peaks, i.e. $\Delta \omega =\omega _{\text {A}_\text {1g}}-\omega _{\text {E}_\text {2g}^1}$. The corresponding emission energies for the 2 L are also indicated with solid orange points.

Previous investigation of PL spectra of artificially twisted BLs MoS$_2$ were carried out at room temperature ^32,39,42^. The PL spectroscopy at low temperature may provide however a more accurate analysis, which is due to the much lower emission linewidths ^4,43^. The PL spectra of the selected BLs with twist angles of 62$^\circ$, 18$^\circ$, and 6$^\circ$ accompanied with those of 1 L and 2 L MoS$_2$ measured at low temperature (*T* = 5 K) are shown in Fig. 3a. Similarly to the aforementioned analysis of the RS, we begin with an examination of the PL spectra of the natural 1 L and 2 L MoS$_2$. The 1 L spectrum consists of two narrow emission lines apparent in the vicinity of the optical band gap (so-called A exciton), which can be ascribed to the neutral and charged excitons in accordance with previous reports ^44–46^. In contrast, the PL spectrum of the 2 L MoS$_2$ is composed of two distinct emission bands: (1) the transitions in the vicinity of the direct A (X$^\text {A}$) and B (X$^\text {B}$) excitons formed at the K$^\pm$ points of the Brillouin zone (BZ), which are observed correspondingly at about 1.93 eV and 2.07 eV; (2) the significantly much intense transitions, denoted as I and I’, apparent at about 1.5 eV, which are ascribed correspondingly to an indirect recombination process between the $\Lambda$ and K point in the conduction band (CB) and $\Gamma$ points in the valence band (VB) of the BZ ^32,43,47^. The PL spectra of the artificial twisted BLs also comprise two direct- and indirect-related bands. While the energies of direct transitions are hardly affected by the twist angles, the energies of the indirect ones change significantly by about 150 meV. The low-intensity emission bands, denoted with * and apparent between the aforementioned I and X$^\text {A}$ lines, can be described as a remaining part of the defect-related emission, which is reported commonly for 1-L MoS$_2$ exfoliated on Si/SiO$_2$ substrates ^44–46^.

To appreciate the effect of the twist angle on the PL spectra, the energy evolution of the X$^\text {A}$, X$^\text {B}$ and I transitions for all the studied samples as a function of twist angle is presented in Fig. 3b. As can be seen in the Figure, there is no effect of the twist angle on the X$^\text {A}$ and X$^\text {B}$ energies. This reflects a pure two-dimensional character of both the A and B excitons, i.e. these complexes are distributed spatially within a single layer even in a bulk form of MoS$_2$ ^48–50^. For the indirect transitions, we focus only on the I emission lines as they are observed for all studied samples. The emission reaches the lowest energies for border cases (6$^\circ$ and 62$^\circ$), which are similar to the value obtained for the natural 2 L MoS$_2$. For BLs with other twist angles, the I energies are substantially higher by about 150 meV and they are almost independent of twist angle. This effect can be associated with changes in the interlayer distance between MLs forming the studied BLs as a function of twist angle, as it was reported previously in Refs. ^32,39^.

Note that the values of I energy and $\Delta \omega$ parameter for BL with twist angle of about 30$^\circ$ are considerably smaller as compared to the corresponding values obtained for other angles within the range 6$^\circ< \Theta <60 ^\circ$ (see Figs. 2c, 3b). It is surprising as both the results indicate opposite cases: the weaker/stronger coupling between ML from the RS/PL results, but it requires more sophisticated theoretical analysis devoted to the effect of twist angles and is out of the scope of this work.

As the energy separation between the A$_\text {1g}$ and E$_\text {2g}^1$ peaks ($\Delta \omega$) can be considered as a probe of the interlayer distance in the studied artificial MoS$_2$ BLs, one can plot the energy dependence of the indirect transition I as a function of $\Delta \omega$ in Fig. 3c. The presented results can be divided into two groups: (1) for structures with twist angle close to 0$^\circ$ and 60$^\circ$, i.e. $\Delta \omega \sim$23 cm$^{-1}$, the I emission energy equals approx. 1.5 eV; (2) for samples with twist angle significantly different from 0$^\circ$ and 60$^\circ$, i.e. $\Delta \omega \sim$22 cm$^{-1}$, the energy dispersion of the I line is of about 80 meV centered at $\sim$1.58 eV. These results are consistent with previously obtained for stacked triangle-shaped MoS$_2$ BLs grown on Si/SiO$_2$ substrate using CVD technique^32^. Summarizing, we can assume that the analysis of the indirect emission versus the $\Delta \omega$ can be used as a useful tool to probe the interlayer distance in the twisted homo-BLs. This allows to distinguish between two cases of twist angles, which are in the vicinity of 0$^\circ$ and 60$^\circ$ or the twist angle is in-between.

**Figure 4:**
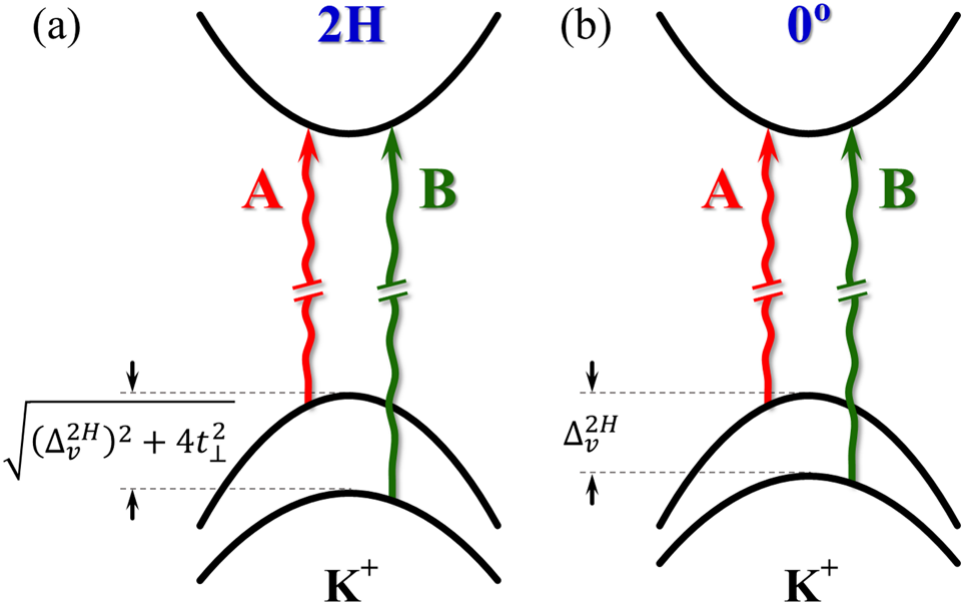
Diagram of relevant subbands in the CB and VB at the K$^+$ point of the Brillouin zone in the bilayers MoS$_2$ with twist angle of (**a**) 60$^\circ$ (2H) and (**b**) 0$^\circ$. The red and green wavy lines show the A and B exciton transitions. The $t_\perp$ and $\Delta ^\text {2H}_v$ represent the interlayer hopping term and the spin-orbit VB splitting without interlayer coupling, respectively.

While the X$^\text {A}$ and X$^\text {B}$ lines are observed for all studied samples, there is an additional emission line, labelled IL, apparent between the former ones for the 2 L and 62$^\circ$ samples, which emission was not reported so far. According to recent results of reflectance contrast (RC) experiments carried out on natural 2H MoS$_2$ BLs at low temperature^51–53^, that line can be associated with the recombination of the so-called interlayer A exciton. The IL line originates from the hybridization of electronic states in the VB of the 2H-stacked BL due to the interlayer coupling (see Ref. ^51^ for details). It is important to mention that the observation of the IL emission is not strictly limited to the 2H phase, reported so far in Refs.^51–53^. As the IL emission is also observed in PL spectra measured on the BL with 62$^\circ$, it indicates that the IL exciton is also present in twisted bilayers with angles varying slightly from exact 60$^\circ$. It is similar to the case of hybridized excitons reported in MoSe$_2$/WS$_2$ heterobilayers ^23^.

The strength of the related VB coupling is described by the interlayer hopping term, $t_\perp$. Based on a **kp** model of 2H BLs in the vicinity of $\mathrm {K}^\pm$ points ^51^, the $t_\perp$ parameter is given by:

1 $$\begin{aligned} t_\perp =\frac{1}{2}\sqrt{(\Delta ^\text {2H}_\text {A-B})^2-(\Delta ^\text {2H}_v)^2}, \end{aligned}$$

where $\Delta ^\text {2H}_\text {A-B}$ is the A-B energy difference for natural 2H BL and $\Delta ^\text {2H}_v$ represents the spin-orbit VB splitting without interlayer coupling. Note that the detailed investigation of the band structure of the 2H and 0$^\circ$ BLs in the vicinity of the K$^\pm$ point of the BZ is performed in Supporting Information. According to that analysis, we assume that: (1) the $\Delta ^\text {2H}_v$ is an order of magnitude larger than its counterpart in the CB $\Delta ^\text {2H}_c$ ^54^; (2) the binding energies of the A and B excitons are comparable; (3) the $\Delta ^\text {2H}_v$ can be roughly approximated by the A-B energy difference for the BL with twist angle of 0$^\circ$. Consequently, the diagram of relevant subbands at the K$^+$ point of the BZ in the MoS$_2$ BLs with twist angle of 60$^\circ$ (2H) and 0$^\circ$ is shown in Fig. 4. As can be appreciated from the Figure, the $t_\perp$ parameter can be evaluated using the energy separation between the A and B excitons measured in the 60$^\circ$ (2H) and 0$^\circ$ BLs. The extracted $\Delta ^\text {2H}_\text {A-B}\sim$180 meV (the corresponding $\Delta ^{62^\circ }_\text {A-B}$ is about 10 meV smaller, which suggests the lower strength of interlayer coupling in twisted BL) and $\Delta ^{6^\circ }_\text {A-B}\sim$150 meV are in very good agreement with the reported ones for natural BLs^51–53^. Using these values, the extracted interlayer hopping parameter ($t_\perp$) is found to be on the order of 50 meV, which agrees very well with the value obtained using the RC experiment performed on the MoS$_2$ BLs grown using CVD technique and encapsulated in hBN flakes (49 meV) ^53^.

**Figure 5:**
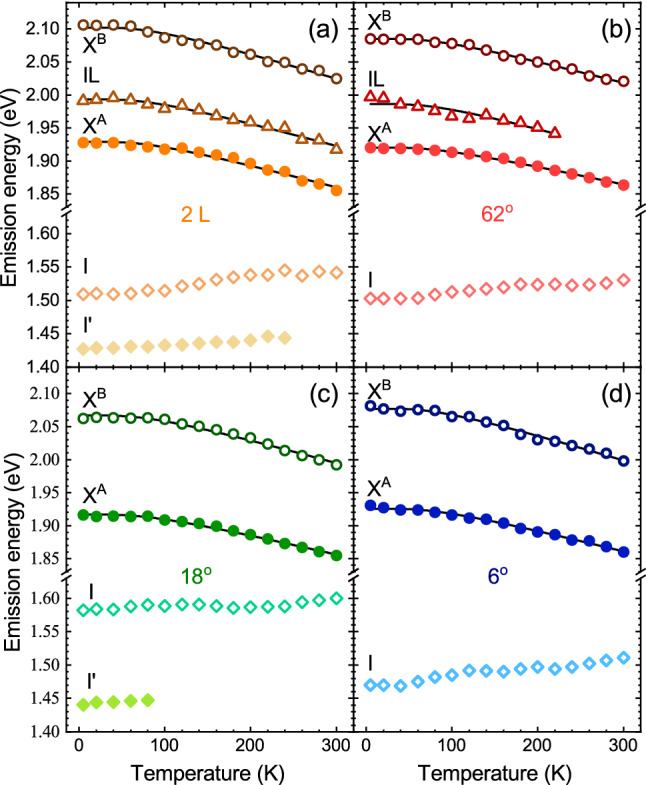
Temperature evolution of the energies of the transitions extracted from PL spectra measured on MoS$_2$: (**a**) bilayer (2 L) and selected homobilayers with twist angles of (**b**) 62$^\circ$, (**c**) 18$^\circ$ and (**d**) 6$^\circ$. The solid black curves are fits to the data obtained with the aid of Odonnell’s formula.

The energy evolution of both the direct (X$^\text {A}$, X$^\text {B}$, and IL) and indirect (I and I’) lines as a function of temperature measured on BL (2 L) and selected homobilayers with twist angles of 6$^\circ$, 18$^\circ$ and 62$^\circ$ is shown in Fig. 5. As can be appreciated in the Figure, all lines within the direct- and indirect-related transitions located at $\sim$ 2.0 eV and $\sim$ 1.5 eV are characterized by the same type of evolution upon increasing temperature, i.e. redshift and blueshift, respectively. The X$^\text {A}$, X$^\text {B}$, and IL peaks redshift when temperature is increased from 5 to 300 K. This can be associated with the reduction of the direct band gap resulting from the temperature expansion of those layers in lateral directions. Consequently, these evolutions can described by the relation proposed by O’Donnell et al.^55^, which expresses the temperature dependence of the band gap in terms of the average energy of acoustic phonons involved in the electron-phonon interaction $\langle \hbar \omega \rangle$. The relation reads $E(T)=E_0-S \langle \hbar \omega \rangle [ \text {coth} ( \langle \hbar \omega \rangle / 2k_{\text {B}} T )-1 ]$, where $E_0$ stands for the band gap at absolute zero temperature, *S* is the coupling constant, and $k_\text {B}$ denotes the Boltzmann constant. We found that $\langle \hbar \omega \rangle$ stayed on nearly the same level, $\sim 21$ meV, for the X$^\text {A}$, X$^\text {B}$, and IL transitions in 2H BL ^47^. As a consequence, we kept it fixed during fittings of all the experimental data shown in Fig. 5. It can be seen that the fitted curves correctly reproduce the energies of excitonic lines (see Fig. 5), which suggests that binding energies of investigated complexes do not depend on temperature. Interestingly, the overall redshift of X$^\text {A}$ lines are of about 60–70 meV, while the corresponding shift for the MoS$_2$ BL exfoliated on Si/SiO$_2$ substrate was found to be of almost 90 meV ^43^. Simultaneously, the crystal expansion across the layers leads to a larger separation between the layers, which results in the blueshift of the indirect band gap. The I emission lines experience almost monotonic blueshifts in the range of about 20–40 meV, while the shift of $\sim$ 80 meV is reported in Ref. ^43^. Both these results indicate that the hBN encapsulation of the BLs induces much smaller expansion of the flake in both directions (in lateral directions and across the layers) with increasing temperature as compared with the BLs exfoliated on Si/SiO$_2$ substrate ^43^.

**Figure 6:**
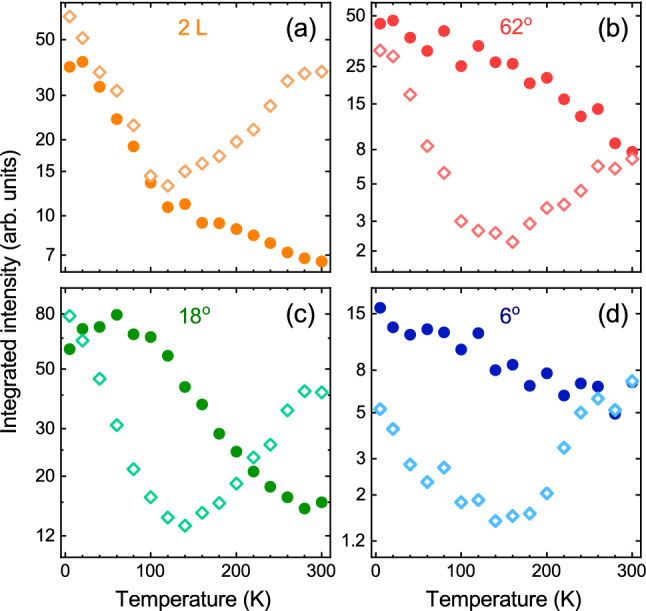
Temperature evolution of the integrated intensities of the (solid circular points) direct- and (open diamond points) indirect-related emissions extracted from PL spectra measured on MoS$_2$: (**a**) bilayer (2 L) and selected homobilayers with twist angles of (**b**) 62$^\circ$, (**c**) 18$^\circ$ and (**d**) 6$^\circ$. Note that the vertical scales are logarithmic.

The temperature evolution of the integrated intensities of the direct- and indirect-related transitions is presented in Fig. 6. It can be seen that the total intensities of all direct transitions mostly decrease monotonically with increasing temperature. The observed trends are in agreement with previous reports for direct emissions apparent in MoS$_2$ ML ^47,56^ and probably result from the competition between the efficiencies of the radiative and non-radiative recombination channels. Surprisingly, the temperature dependencies of the corresponding indirect transition intensities are clearly non-monotonic. First, quick reductions of the related intensities is observed from 5 K to about 100–150 K, which can be associated with increase of kinetic energies of excitons and interplay between radiative and non-radiative processes. Then it is followed by slower increase of the intensities up to room temperature, which can be explained in terms of the increased population of phonons at higher temperatures.

## Summary

A systematic investigation of optical properties of hBN-encapsulated artificially stacked MoS$_2$ BLs with varying interlayer twist angle has been conducted. It has been shown that RS spectroscopy is a sensible tool for determining the interlayer coupling and spacing in the BL. The strongest coupling has been observed in MoS$_2$ homobilayers with twist angles of 6$^{\circ }$ and 62$^{\circ }$, which reproduce well the structure of 2H and 3R polytypes found in natural and CVD-grown MoS$_2$ multilayers. Increasing the twist angle of homobilayers from 6$^{\circ }$ to $\sim 30^{\circ }$ leads to an increase in the interlayer spacing, and thus a decrease in the interlayer coupling. This effect can be observed as the absence of the low-energy interlayer phonon modes in the RS spectrum as well as the energy shift of $\sim 140$ meV for the indirect transition between K-$\Gamma$ points of the Brillouin zone. The PL of artificially assembled homobilayers, in comparison with the natural 2 L MoS$_2$, features a single line associated with indirect emission, but its energy varies considerably depending on the interlayer twist. We conclude that gaining a new degree of freedom by inter-layer twisting of artificially assembled flakes permits the control over the energy of the indirect transition, This offers further insight in few-layer 2D systems and can be a useful tool for future device applications.

## Methods

The investigated MoS$_2$ BLs, MLs, and hBN flakes were fabricated by two-stage PDMS-based mechanical exfoliation of bulk crystals. An unoxidized Si wafer was used as a substrate. In order to ensure the best quality of the substrate surface, they were annealed at 200 $^{\circ }$C and kept on hot-plate until the first non-deterministic transfer of h-BN flakes. Subsequent layers were transferred deterministically, to reduce inhomogeneities between each transfer sample were annealed. The complete structures were annealed at 160 $^{\circ }$C for 1.5 h to ensure the best layer-to-layer and layer-to-substrate adhesion and to eliminate a substantial portion of air pockets on the interfaces between the constituent layers.

The SHG measurements performed to identify the relative twist angle of the exfoliated S-TMD MLs forming homobilayers were taken at $T=300$ K using a home-built setup with a femtosecond Ti:Sapphire laser with excitation at 800 nm (1.55 eV). For each measurement, the laser light had a typical incident power of 500 $\mu$W, was linearly polarized, and focused to a spot size of 1 $\mu$m by a 50x objective lens. A set of a motorized half-wave plate and a fixed linear polarizer were used to analyse an SHG signal, which was detected by a Si avalanche photodiode.

The PL and RS measurements were performed using $\lambda$ = 515 nm (2.41 eV) radiation from continuous-wave Ar-ion or diode lasers. For the PL and RS experiments at room temperature ($T=300$ K), the excitation light was focused through a 100x long-working distance objective with a 0.55 numerical aperture (NA) producing a spot of about 1 $\mu$m diameter. The signal was collected via the same microscope objective, sent through a 1 m monochromator, and then detected by using a liquid nitrogen-cooled charge-coupled device (CCD) camera. To detect low-energy RS up to about ±10 cm$^{-1}$ from the laser line, a set of Bragg filters was implemented in both excitation and detection paths. The temperature-dependent PL measurements were performed using an analogous setup with small modifications (a 50$\times$ long-working distance objective and a 0.5 m monochromator). Moreover, the studied samples were placed on a cold finger in a continuous flow cryostat mounted on *x*–*y* motorized positioners. The excitation power focused on the sample was kept at 200 $\mu$W during all measurements to avoid local heating.

## Supplementary Information

Supplementary Information 1.

## Data Availability

The datasets obtained during experiments and analysis in course of manuscript preparation are available from the corresponding author on reasonable request.
