# Supplementary material for: The optical response of artificially twisted MoS2 bilayers
Source: Sci Rep. 2021 Aug 23;11:17037. doi: 10.1038/s41598-021-95700-5 (PMC8382769; doi:10.1038/s41598-021-95700-5)
Supplement: Supplementary file 1 — Supplementary Information 1. [file 41598_2021_95700_MOESM1_ESM.pdf]

Supporting Information
The optical response of artificially twisted MoS_2 bilayers

M. Grzeszczyk,[1,*] J. Szpakowski,[1] A. O. Slobodeniuk,[2] T. Kazimierczuk,[1] M. Bhatnagar,[1] T. Taniguchi,[3] K. Watanabe,[4] P. Kossacki,[1] M. Potemski,[1,5] A. Babiński,[1] and M. R. Molas[1,†]

[1]*Institute of Experimental Physics, Faculty of Physics, University of Warsaw, ul. Pasteura 5, 02-093 Warsaw, Poland*
[2]*Department of Condensed Matter Physics, Faculty of Mathematics and Physics, Charles University, Ke Karlovu 5, Praha 2 CZ-121 16, Czech Republic*
[3]*International Center for Materials Nanoarchitectonics, National Institute for Materials Science, 1-1 Namiki, Tsukuba 305-0044, Japan*
[4]*Research Center for Functional Materials, National Institute for Materials Science, 1-1 Namiki, Tsukuba 305-0044, Japan*
[5]*Laboratoire National des Champs Magnétiques Intenses, CNRS-UGA-UPS-INSA-EMFL, 25, avenue des Martyrs, 38042 Grenoble, France*

I. BILAYER WITH 60°-ANGLE ALIGNMENT

We model a S-TMD bilayer with 60°-angle alignment as a pile of two monolayers (top and bottom), placed in parallel to xy plane. We define the positions of metal and chalcogen atoms of the bottom layer as

$$\mathbf{R}_{mn}^{M,b} = \mathbf{a}_1 m + \mathbf{a}_2 n + \mathbf{t}_M = \mathbf{R}_{mn} + \mathbf{t}_M, \tag{1}$$

$$\mathbf{R}_{mn}^{X_\pm,b} = \mathbf{a}_1 m + \mathbf{a}_2 n + \mathbf{t}_X \pm \eta \mathbf{e}_z = \mathbf{R}_{mn} + \mathbf{t}_X \pm \eta \mathbf{e}_z, \tag{2}$$

respectively. Here we introduced the in-plane primitive lattice vectors of length a_0

$$\mathbf{a}_1 = \frac{a_0}{2}(\mathbf{e}_x + \sqrt{3}\mathbf{e}_y), \quad \mathbf{a}_2 = \frac{a_0}{2}(\mathbf{e}_x - \sqrt{3}\mathbf{e}_y), \tag{3}$$

the pair of integer numbers (m, n), the short notation for the (m, n)-th lattice vector $\mathbf{R}_{mn} = \mathbf{a}_1 m + \mathbf{a}_2 n$ and unit vectors of Cartesian coordinate system $\mathbf{e}_x, \mathbf{e}_y, \mathbf{e}_z$. Vectors

$$\mathbf{t}_M = \frac{a_0}{2}\Big(\mathbf{e}_x + \frac{1}{\sqrt{3}}\mathbf{e}_y\Big), \quad \mathbf{t}_X = \frac{a_0}{2}\Big(\mathbf{e}_x - \frac{1}{\sqrt{3}}\mathbf{e}_y\Big) \tag{4}$$

define in-plane positions of the metal and chalcogen atoms within a unit cell of S-TMD monolayer, respectively. Vectors $\pm\eta\mathbf{e}_z$ with $\eta > 0$ define the out-of-plane positions of chalcogen atoms in the unit cell. We also introduce the primitive vectors of reciprocal lattice

$$\mathbf{b}_1 = \frac{2\pi}{a_0}\Big(\mathbf{e}_x + \frac{1}{\sqrt{3}}\mathbf{e}_y\Big), \quad \mathbf{b}_2 = \frac{2\pi}{a_0}\Big(\mathbf{e}_x - \frac{1}{\sqrt{3}}\mathbf{e}_y\Big). \tag{5}$$

They satisfy the orthogonality property $\mathbf{a}_j \mathbf{b}_k = 2\pi\delta_{jk}$, where δ_{jk} is the Kronecker delta.

The top lattice of the bilayer can be obtained from the bottom one as a result of the shift along z direction on some distance l with subsequent rotation around Oz axis on 180° degree. Then, the positions of the metal and chalcogen atoms of the top lattice become

$$\mathbf{R}_{mn}^{M,t} = \mathbf{R}_{mn} + l\mathbf{e}_z + \mathbf{t}_X, \tag{6}$$

$$\mathbf{R}_{mn}^{X_\pm,t} = \mathbf{R}_{mn} + l\mathbf{e}_z + \mathbf{t}_M \pm \eta\mathbf{e}_z. \tag{7}$$

Note that all of the chalcogen atoms of bottom layer are aligned with the metal atoms of the top layer (and vice versa) along z-direction. Hence, the unit cell of the bilayer contains twice more atoms than in 1 L. The positions of metal and chalcogen atoms within the unit cell are defined by vectors $\{\mathbf{t}_M, \mathbf{t}_X + l\mathbf{e}_z\}$ and $\{\mathbf{t}_X \pm \eta\mathbf{e}_z, \mathbf{t}_M \pm \eta\mathbf{e}_z + l\mathbf{e}_z\}$, respectively. This arrangement of atoms is called 2H-stacking and corresponds to thermodynamically stable form of all S-TMD crystals with any number of layers including bulk.

The bilayer possesses C_3 rotation symmetry (with Oz line as the rotational axis) and mirror symmetry $P : x \leftrightarrow -x$ (the mirror's plane is yz-plane). Therefore, the crystal has the same hexagonal Brillouin zone as the Brillouin zone of the bottom layer. Hence, it is convenient to use the known Bloch states of the monolayer as the basis states. Namely we are interested in conduction (c) and valence (v) band states at the $K^\pm$ points. To this end, we introduce the vectors $\pm\mathbf{K} = \pm(\mathbf{b}_1 + \mathbf{b}_2)/3$, which define the position of the $K^\pm$ points in the reciprocal space, respectively. In

further we will use the notation $\pm\mathbf{K}$ both for vectors and the positions of the edges of the Brillouin zone ($\mathrm{K}^{\pm}$), for clarity. In the vicinity of $\pm\mathbf{K}$ points the Bloch states are predominantly made of the d-orbitals of metal atoms. The corresponding valence and conduction bands states of the bottom layer can be presented as

$$\Psi^b_{\pm\mathbf{K},v}(\mathbf{r}) = \frac{1}{\sqrt{N}} \sum_{\mathbf{R}^{M,b}_{mn}} e^{\pm i\mathbf{K}\mathbf{R}^{M,b}_{mn}} Y_{2,\pm 2}(\mathbf{r}-\mathbf{R}^{M,b}_{mn}), \tag{8}$$

$$\Psi^b_{\pm\mathbf{K},c}(\mathbf{r}) = \frac{1}{\sqrt{N}} \sum_{\mathbf{R}^{M,b}_{mn}} e^{\pm i\mathbf{K}\mathbf{R}^{M,b}_{mn}} Y_{2,0}(\mathbf{r}-\mathbf{R}^{M,b}_{mn}). \tag{9}$$

Here N is the normalization factor and $Y_{lm}(\mathbf{r}-\mathbf{R})$ is the value of the lm-th atomic orbital placed at the point $\mathbf{R}$ and calculated at the point $\mathbf{r}$. The operator $\widehat{C}_3$, which generates $R_{2\pi/3}$ rotation of the vectors in space, transforms the corresponding Bloch functions as

$$\begin{aligned}
\widehat{C}_3\Psi^b_{\pm\mathbf{K},v}(\mathbf{r}) =& \frac{1}{\sqrt{N}} \sum_{\mathbf{R}_{mn}} e^{\pm i\mathbf{K}(\mathbf{R}_{mn}+\mathbf{t}_M)} Y_{2,\pm 2}(R^{-1}_{2\pi/3}\mathbf{r}-\mathbf{R}_{mn}-\mathbf{t}_M) = \\
=& e^{\mp 4\pi i/3}\frac{1}{\sqrt{N}} \sum_{\mathbf{R}_{mn}} e^{\pm i\mathbf{K}(\mathbf{R}_{mn}+\mathbf{t}_M)} Y_{2,\pm 2}(\mathbf{r}-R_{2\pi/3}[\mathbf{R}_{mn}+\mathbf{t}_M]) = \\
=& e^{\mp 4\pi i/3}\frac{1}{\sqrt{N}} \sum_{\mathbf{R}_{m'n'}} e^{\pm iR_{2\pi/3}\mathbf{K}(\mathbf{R}_{m'n'}+\mathbf{t}_M)} Y_{2,\pm 2}(\mathbf{r}-\mathbf{R}_{m'n'}-\mathbf{t}_M) = \\
=& e^{\mp 4\pi i/3}e^{\mp i\mathbf{b}_2\mathbf{t}_M}\frac{1}{\sqrt{N}} \sum_{\mathbf{R}_{mn}} e^{\pm i\mathbf{K}(\mathbf{R}_{mn}+\mathbf{t}_M)} Y_{2,\pm 2}(\mathbf{r}-\mathbf{R}_{mn}-\mathbf{t}_M) = \Psi^b_{\pm\mathbf{K},v}(\mathbf{r}),
\end{aligned} \tag{10}$$

$$\widehat{C}_3\Psi^b_{\pm\mathbf{K},c}(\mathbf{r}) = e^{\mp 2\pi i/3}\Psi^b_{\pm\mathbf{K},c}(\mathbf{r}). \tag{11}$$

The phases, which appear under the transformation of the basis states of the bottom layer correspond to the notation in Ref. 1.

In addition, the crystal has inversion symmetry $I : \mathbf{r} \leftrightarrow -\mathbf{r} + 2\mathbf{R}_I$, with the center of inversion in the point $\mathbf{R}_I = l\mathbf{e}_z/2$. This symmetry together with the time-reversal symmetry induces the restriction on the band structure of the crystal. In accordance to the Kramers theorem, all the bands of the crystal become doubly degenerated by spin. Therefore, it is convenient to define the second pair of basis states, associated with the top layer, using the above-mentioned symmetry operations $\Psi^t_{\pm\mathbf{K},n}(\mathbf{r}) = \widehat{K}_0\widehat{I}\Psi^b_{\pm\mathbf{K},n}(\mathbf{r})$. Here $\widehat{K}_0$ and $\widehat{I}$ are complex conjugation and inversion symmetry operators, respectively. Using the tight-binding representation of the basis states of the bottom layer we get

$$\begin{aligned}
\Psi^t_{\pm\mathbf{K},v}(\mathbf{r}) = \widehat{K}_0\widehat{I}\Psi^b_{\pm\mathbf{K},v}(\mathbf{r}) =& \frac{1}{\sqrt{N}} \sum_{\mathbf{R}_{mn}} e^{\mp i\mathbf{K}(\mathbf{R}_{mn}+\mathbf{t}_M)} Y^*_{2,\pm 2}(I^{-1}\mathbf{r}-\mathbf{R}_{mn}-\mathbf{t}_M) = \\
=& \frac{1}{\sqrt{N}} \sum_{\mathbf{R}_{mn}} e^{\mp i\mathbf{K}(\mathbf{R}_{mn}+\mathbf{t}_M)} Y_{2,\mp 2}(\mathbf{r}-I[\mathbf{R}_{mn}+\mathbf{t}_M]) = \\
=& \frac{1}{\sqrt{N}} \sum_{\mathbf{R}_{m'n'}} e^{\mp iI\mathbf{K}(\mathbf{R}_{m'n'}+\mathbf{t}_X)} Y_{2,\mp 2}(\mathbf{r}-\mathbf{R}_{m'n'}-\mathbf{t}_X-l\mathbf{e}_z) = \\
=& \frac{1}{\sqrt{N}} \sum_{\mathbf{R}_{mn}} e^{\pm i\mathbf{K}(\mathbf{R}_{mn}+\mathbf{t}_X)} Y_{2,\mp 2}(\mathbf{r}-\mathbf{R}_{mn}-\mathbf{t}_X-l\mathbf{e}_z),
\end{aligned} \tag{12}$$

$$\Psi^t_{\pm\mathbf{K},c}(\mathbf{r}) = \widehat{K}_0\widehat{I}\Psi^b_{\pm\mathbf{K},c}(\mathbf{r}) = \frac{1}{\sqrt{N}} \sum_{\mathbf{R}_{mn}} e^{\pm i\mathbf{K}(\mathbf{R}_{mn}+\mathbf{t}_X)} Y_{2,0}(\mathbf{r}-\mathbf{R}_{mn}-\mathbf{t}_X-l\mathbf{e}_z). \tag{13}$$

The states satisfy the following transformation rules under rotation $\widehat{C}_3\Psi^t_{\pm\mathbf{K},v}(\mathbf{r}) = \Psi^t_{\pm\mathbf{K},v}(\mathbf{r})$, $\widehat{C}_3\Psi^t_{\pm\mathbf{K},c}(\mathbf{r}) = e^{\pm 2\pi i/3}\Psi^t_{\pm\mathbf{K},c}(\mathbf{r})$, with phases which are opposite to the phases of the basis states of the bottom layer $\widehat{C}_3\Psi^b_{\pm\mathbf{K},v}(\mathbf{r}) = \Psi^b_{\pm\mathbf{K},v}(\mathbf{r})$, $\Psi^b_{\pm\mathbf{K},c}(\mathbf{r}) = e^{\mp 2\pi i/3}\Psi^b_{\pm\mathbf{K},c}(\mathbf{r})$. It leads to the fact that bilayer crystal can absorb the light with both circular polarizations in $\mathbf{K}$ point as well as in $-\mathbf{K}$ one. This feature is a consequence of inversion symmetry of the crystal. Finally, the mirror symmetry operator $\widehat{P}$ acts on the basis states as $\widehat{P}\Psi^\alpha_{\pm\mathbf{K},n}(\mathbf{r}) = [\Psi^\alpha_{\pm\mathbf{K},n}(\mathbf{r})]^* = \Psi^\alpha_{\mp\mathbf{K},n}(\mathbf{r})$, where $n = c, v$ and $\alpha = b, t$.

In further we focus on the states at the $\mathbf{K}$ point for brevity. We take into account the spin degree of freedom $s =\uparrow,\downarrow$ of electron excitations and introduce the following set of 8 basis states

$$|\Psi_c^b, s\rangle = \Psi_{\mathbf{K},c}^b(\mathbf{r})|s\rangle, \quad |\Psi_v^b, s\rangle = \Psi_{\mathbf{K},v}^b(\mathbf{r})|s\rangle, \tag{14}$$

$$|\Psi_c^t, s\rangle = \Psi_{\mathbf{K},c}^t(\mathbf{r})|s\rangle, \quad |\Psi_v^t, s\rangle = \Psi_{\mathbf{K},v}^t(\mathbf{r})|s\rangle. \tag{15}$$

According to the $\mathbf{kp}$ method, developed for S-TMD multilayers[2–4] the quasiparticles with the momentum $\mathbf{k} = k_x\mathbf{e}_x + k_y\mathbf{e}_y$ at the $\mathbf{K}$ point are described by the matrix elements $\langle\Psi_n^\alpha, s|\widehat{H}|\Psi_{n'}^{\alpha'}, s'\rangle$ of the one-particle Hamiltonian

$$H(\mathbf{r}) = \frac{\widehat{\mathbf{p}}^2}{2m_0} + U^b(\mathbf{r}) + U^t(\mathbf{r}) + \frac{\hbar}{4m_0^2c^2}\left[\nabla U^b(\mathbf{r}), \widehat{\mathbf{p}}\right]\boldsymbol{\sigma} + \frac{\hbar}{4m_0^2c^2}\left[\nabla U^t(\mathbf{r}), \widehat{\mathbf{p}}\right]\boldsymbol{\sigma} + \frac{\hbar}{m_0}\mathbf{k}\widehat{\mathbf{p}}. \tag{16}$$

Here m_0 is electron's mass, c – speed of light, $\hbar$ – Planck's constant, $\boldsymbol{\sigma} = (\sigma_x, \sigma_y, \sigma_z)$ are Pauli matrices and $\widehat{\mathbf{p}} = -i\hbar\nabla$ is the momentum operator. The first term of the Hamiltonian defines the kinetic energy of an electron which propagates in the crystal field of the bottom $U^b(\mathbf{r})$ and top $U^t(\mathbf{r})$ layers of the bilayer. The next two terms describe the spin-orbital interaction in the system, induced by the potentials $U^b(\mathbf{r})$ and $U^t(\mathbf{r})$, respectively. The last $\mathbf{kp}$ term couples valence and conduction bands. This coupling is supposed to be small and we omit its effects for the current study. The detailed analysis of the impact of the $\mathbf{kp}$ term can be found in Refs. 3,4.

We consider first the matrix elements of the states of bottom layer. We present the Hamiltonian as

$$H(\mathbf{r}) = H_0^b(\mathbf{r}) + H_{int}^t(\mathbf{r}), \tag{17}$$

where we introduced the Hamiltonian of the bottom monolayer

$$H_0^b(\mathbf{r}) = \frac{\widehat{\mathbf{p}}^2}{2m_0} + U^b(\mathbf{r}) + \frac{\hbar}{4m_0^2c^2}\left[\nabla U^b(\mathbf{r}), \widehat{\mathbf{p}}\right]\boldsymbol{\sigma}, \tag{18}$$

and the term which affects the motion of quasiparticles of the bottom layer by the crystal field of the top layer

$$H_{int}^t(\mathbf{r}) = U^t(\mathbf{r}) + \frac{\hbar}{4m_0^2c^2}\left[\nabla U^t(\mathbf{r}), \widehat{\mathbf{p}}\right]\boldsymbol{\sigma}. \tag{19}$$

The Hamiltonian $H_0^b(\mathbf{r})$ has the diagonal matrix elements,which are nothing but the position of the conduction and valence bands in monolayer

$$\langle\Psi_v^b, s|H_0^b(\mathbf{r})|\Psi_v^b, s\rangle = E_v + \sigma_s\Delta_v/2, \tag{20}$$

$$\langle\Psi_c^b, s|H_0^b(\mathbf{r})|\Psi_c^b, s\rangle = E_c + \sigma_s\Delta_c/2, \tag{21}$$

Here E_v and E_c are positions of the valence and conduction bands without spin splitting, Δ_v and Δ_c are their spin splittings, and $\sigma_s = +1(-1)$ for $s =\uparrow$ ($\downarrow$) states respectively. Note that in $\mathbf{K}$ point Δ_v is always positive, while Δ_c can be negative (bright type of S-TMD) and positive (darkish type of S-TMD).

The dominant contribution from of $H_{int}^t(\mathbf{r})$ is the diagonal matrix elements in spin space

$$\langle\Psi_v^b, s|H_{int}^t(\mathbf{r})|\Psi_v^b, s\rangle = \delta E_v + \sigma_s\delta\Delta_v/2, \tag{22}$$

$$\langle\Psi_c^b, s|H_{int}^t(\mathbf{r})|\Psi_c^b, s\rangle = \delta E_c + \sigma_s\delta\Delta_c/2. \tag{23}$$

We suppose that the corrections to the splitting are small $|\Delta_c| \gg |\delta\Delta_c|$, $|\Delta_v| \gg |\delta\Delta_v|$, and the type of S-TMD bilayer remains the same as the type of its constituents. $H_{int}^t(\mathbf{r})$ term has also non-zero matrix element between valence and conduction bands of the same layer and opposite spins (see Refs. 5,6 for details). However, these matrix elements give the negligibly small contribution to the energies of the bands, proportional to $\sim 1/(E_c - E_v)$. Therefore, we omit them from the study.

The matrix elements between the states of the top layer can be calculated in the same way. We present the total Hamiltonian as

$$H(\mathbf{r}) = H_0^t(\mathbf{r}) + H_{int}^b(\mathbf{r}), \tag{24}$$

where the first term

$$H_0^t(\mathbf{r}) = \frac{\widehat{\mathbf{p}}^2}{2m_0} + U^t(\mathbf{r}) + \frac{\hbar}{4m_0^2c^2}\left[\nabla U^t(\mathbf{r}), \widehat{\mathbf{p}}\right]\boldsymbol{\sigma}, \tag{25}$$

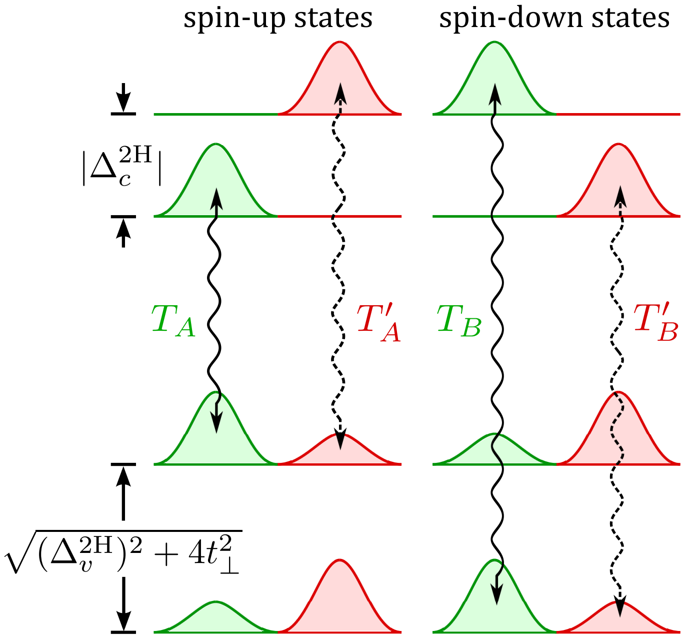

FIG. 1. The bands positions and optical transitions in the $+\mathbf{K}$ point of 2H stacked MoS_2 bilayer. Left/right side represents the spin-up/spin-down states in the system. Green and red convexes represent the conduction and valence band states associated with optical transitions active in the σ^+ and σ^- polarizations, respectively. The green/red color also denotes the bottom/top layer. Solid (dashed) wavy arrows indicates optical transitions T_A, T_B (T'_A , T'_B) due to the intralayer A and B (interlayer A$'$ and B$'$) excitons. $|\Delta_c^{2H}|$ and $\sqrt{(\Delta_v^{2H})^2 + 4t_\perp^2}$ denote the splitting in the conduction (c) and the valence (v) bands, respectively.

is the Hamiltonian of the top monolayer, while the second term

$$H^b_{int}(\mathbf{r}) = U^b(\mathbf{r}) + \frac{\hbar}{4m_0^2c^2}\left[\nabla U^b(\mathbf{r}), \widehat{\mathbf{p}}\right]\boldsymbol{\sigma}, \tag{26}$$

affects the motion of quasiparticles of the top layer by the crystal field of the bottom one. With the help of Kramers theorem and the latter result one can immediately get the answer for the matrix elements of the above-mentioned terms

$$\langle \Psi^t_v, s|H^t_0(\mathbf{r})|\Psi^t_v, s\rangle = E_v - \sigma_s\Delta_v/2, \tag{27}$$
$$\langle \Psi^t_c, s|H^t_0(\mathbf{r})|\Psi^t_c, s\rangle = E_c - \sigma_s\Delta_c/2, \tag{28}$$
$$\langle \Psi^t_v, s|H^b_{int}(\mathbf{r})|\Psi^t_v, s\rangle = \delta E_v - \sigma_s\delta\Delta_v/2, \tag{29}$$
$$\langle \Psi^t_c, s|H^b_{int}(\mathbf{r})|\Psi^t_c, s\rangle = \delta E_c - \sigma_s\delta\Delta_c/2. \tag{30}$$

Note that the sign before spin-splitting terms for the states of the top layer is opposite to the sign of the same terms of the bottom layer. This is the manifestation of the double degeneracy by spin of all the bands of the bilayer.

Finally, we calculate the matrix elements of the Hamiltonian between the states of the different layers. Namely, we evaluate the following interlayer matrix elements $\langle \Psi^t_n, s|H(\mathbf{r})|\Psi^b_{n'}, s'\rangle$, where $n, n' = c, v$. The other matrix elements can be obtained by complex conjugation of the considered ones. We present the Hamiltonian in the following way

$$H(\mathbf{r}) = H^t_0(\mathbf{r}) + H^b_0(\mathbf{r}) - \frac{\widehat{\mathbf{p}}^2}{2m_0}. \tag{31}$$

and suppose the orthogonality of the states from the opposite layers $\langle \Psi^t_n, s|\Psi^b_{n'}, s'\rangle = 0$. Then

$$\langle \Psi^t_n, s|H(\mathbf{r})|\Psi^b_{n'}, s'\rangle = -\frac{1}{2m_0}\langle \Psi^t_n, s|\widehat{\mathbf{p}}^2|\Psi^b_{n'}, s'\rangle. \tag{32}$$

The $\widehat{\mathbf{p}}^2$ operator is a spin singlet, hence the matrix elements are diagonal in spin space $\langle \Psi^t_n, s|\widehat{\mathbf{p}}^2|\Psi^b_{n'}, s'\rangle = \delta_{ss'}\langle \Psi^t_n|\widehat{\mathbf{p}}^2|\Psi^b_{n'}\rangle$, where $|\Psi^\alpha_n\rangle = \Psi^\alpha_{\mathbf{K},n}(\mathbf{r})$. Using transformation properties of the basis states under C_3 rotation

$$\langle \Psi_v^t | \hat{\mathbf{p}}^2 | \Psi_c^b \rangle = \langle \Psi_v^t | \hat{C}_3^{-1} \hat{C}_3 \hat{\mathbf{p}}^2 \hat{C}_3^{-1} \hat{C}_3 | \Psi_c^b \rangle = e^{-2\pi i/3} \langle \Psi_v^t | \hat{\mathbf{p}}^2 | \Psi_c^b \rangle = 0, \tag{33}$$

$$\langle \Psi_c^t | \hat{\mathbf{p}}^2 | \Psi_c^b \rangle = \langle \Psi_c^t | \hat{C}_3^{-1} \hat{C}_3 \hat{\mathbf{p}}^2 \hat{C}_3^{-1} \hat{C}_3 | \Psi_c^b \rangle = e^{-4\pi i/3} \langle \Psi_c^t | \hat{\mathbf{p}}^2 | \Psi_c^b \rangle = 0, \tag{34}$$

$$\langle \Psi_v^t | \hat{\mathbf{p}}^2 | \Psi_v^b \rangle = \langle \Psi_v^t | \hat{C}_3^{-1} \hat{C}_3 \hat{\mathbf{p}}^2 \hat{C}_3^{-1} \hat{C}_3 | \Psi_v^b \rangle = \langle \Psi_v^t | \hat{\mathbf{p}}^2 | \Psi_v^b \rangle = t_\perp \neq 0, \tag{35}$$

$$\langle \Psi_c^t | \hat{\mathbf{p}}^2 | \Psi_v^b \rangle = \langle \Psi_c^t | \hat{C}_3^{-1} \hat{C}_3 \hat{\mathbf{p}}^2 \hat{C}_3^{-1} \hat{C}_3 | \Psi_v^b \rangle = e^{-2\pi i/3} \langle \Psi_c^t | \hat{\mathbf{p}}^2 | \Psi_v^b \rangle = 0, \tag{36}$$

we get that only one matrix element is non-zero. The P symmetry of the crystal dictates that the parameter $t_\perp$ is a real number $\mathrm{Im}[t_\perp] = 0$. This parameter couples the valence bands of the same spin of the top and bottom layers, mixes them and forms the new type of valence band states, presented in Fig. 1 as two-convex structure. It indicates that the valence band Bloch state in the $+\mathbf{K}$ point of the bilayer is a superposition of the corresponding valence band Bloch states of the bottom and top layers of the same spin. On the contrary, the single-convex representation of the conduction band Bloch states in the $+\mathbf{K}$ point of the bilayer indicates that the corresponding conduction band states of the top and bottom layers are not mixed. As a result, the dipole matrix elements between the new valence band states and conduction band states of the top as well as of the bottom layers become non-zero. It causes to doubling of number of possible optical transition in the system.

Summarizing the aforementioned calculations we conclude that there are four optical transitions in 2H-stacked bilayer: A and B intense optical transitions (which form intralayer exciton complexes) and weak optical transitions A' and B'(which form inter-layer exciton complexes). The energies of these excitons are

$$E_A = -\mathcal{E}_A + E_c^{2\mathrm{H}} - E_v^{2\mathrm{H}} + \frac{\Delta_c^{2\mathrm{H}}}{2} - \frac{\sqrt{(\Delta_v^{2\mathrm{H}})^2 + 4t_\perp^2}}{2}, \tag{37}$$

$$E_B = -\mathcal{E}_B + E_c^{2\mathrm{H}} - E_v^{2\mathrm{H}} - \frac{\Delta_c^{2\mathrm{H}}}{2} + \frac{\sqrt{(\Delta_v^{2\mathrm{H}})^2 + 4t_\perp^2}}{2}, \tag{38}$$

$$E_{A'} = -\mathcal{E}_{A'} + E_c^{2\mathrm{H}} - E_v^{2\mathrm{H}} - \frac{\Delta_c^{2\mathrm{H}}}{2} - \frac{\sqrt{(\Delta_v^{2\mathrm{H}})^2 + 4t_\perp^2}}{2}, \tag{39}$$

$$E_{B'} = -\mathcal{E}_{B'} + E_c^{2\mathrm{H}} - E_v^{2\mathrm{H}} + \frac{\Delta_c^{2\mathrm{H}}}{2} + \frac{\sqrt{(\Delta_v^{2\mathrm{H}})^2 + 4t_\perp^2}}{2}. \tag{40}$$

Here we introduced he absolute values of the binding energies of corresponding excitons $\mathcal{E}_A, \mathcal{E}_B, \mathcal{E}_{A'}, \mathcal{E}_{B'}$, and we the short notations $E_c^{2\mathrm{H}} = E_c + \delta E_c$, $E_v^{2\mathrm{H}} = E_v + \delta E_v$, $\Delta_c^{2\mathrm{H}} = \Delta_c + \delta\Delta_c$ and $\Delta_v^{2\mathrm{H}} = \Delta_v + \delta\Delta_v$. The sketch of the bands position in MoS_2 bilayer with 2H-stacking is presented in Fig. 1.

For the particular case of MoS_2 the splitting in conduction band is supposed to be much smaller than the splitting in valence band $|\Delta_c^{2\mathrm{H}}| \ll |\Delta_v^{2\mathrm{H}}|$, and the binding energies of A and B excitons are considered to be equal $\mathcal{E}_A = \mathcal{E}_B$. In this approximation we have the following result $\Delta_{A-B}^{2\mathrm{H}} = E_B - E_A \approx \sqrt{(\Delta_v^{2\mathrm{H}})^2 + 4t_\perp^2}$.

Note that the intralayer and interlayer optical transitions in the same $\mathbf{K}$ (or $-\mathbf{K}$) point are characterized by opposite circular polarizations and g-factors of corresponding excitons (see Refs. 3,4 for details).

II. BILAYER WITH 0°-ANGLE ALIGNMENT

In order to compare the results of the measurements presented in the main text we describe the optical properties of the bilayer S-TMD with zero-angle alignment (in further "bilayer") in the similar way as it was done for 2H-stacked bilayer. Again, we consider the bilayer as a pile of two monolayers (top and bottom), placed in parallel to xy plane. We assume the positions of metal and chalcogen atoms of the bottom layer are the same as in the previous section

$$\mathbf{R}_{mn}^{M,b} = \mathbf{R}_{mn} + \mathbf{t}_M, \tag{41}$$

$$\mathbf{R}_{mn}^{X_\pm,b} = \mathbf{R}_{mn} + \mathbf{t}_X \pm \eta \mathbf{e}_z, \tag{42}$$

The top lattice of the bilayer can be obtained from the bottom one as a result of two consequent shifts: along z direction on distance l (which is not equal to the distance l for the 2H-stacked bilayer) and then along y direction on distance $a_0/\sqrt{3}$. Then, the position of the metal and chalcogen atoms of the top lattice can be presented as

$$\mathbf{R}_{mn}^{M,t} = \mathbf{R}_{mn} + l\mathbf{e}_z + \mathbf{a}_1, \tag{43}$$

$$\mathbf{R}_{mn}^{X_\pm,t} = \mathbf{R}_{mn} + l\mathbf{e}_z + \mathbf{t}_M \pm \eta \mathbf{e}_z. \tag{44}$$

Note that the half of the chalcogen and half of metal atoms in this bilayer are aligned in z-direction. This type of stacking for hexagonal lattices is called Bernal or AB-stacking. The unit cell of the considering bilayer contains twice more atoms than in monolayer. The positions of metal and chalcogen atoms within the unit cell are defined by vectors $\{\mathbf{t}_M, \mathbf{a}_1 + l\mathbf{e}_z\}$ and $\{\mathbf{t}_X \pm \eta\mathbf{e}_z, \mathbf{t}_M \pm \eta\mathbf{e}_z + l\mathbf{e}_z\}$, respectively.

Note that the considering lattice has neither in-plane mirror symmetry (like AA-stacked case) nor inversion symmetry (like 2H-stacked bilayer). It possesses only C_3 rotation symmetry (with Oz line as a rotational axis) and mirror symmetry $P : x \leftrightarrow -x$ (the mirror's plane is yz-plane). Again the crystal has the same hexagonal Brillouin zone as the Brillouin zone of the bottom layer. Hence, we choose the same basis states for valence and conduction bands in the $\pm\mathbf{K}$ points of the bottom layer as we have in the previous section

$$\Psi^b_{\pm\mathbf{K},v}(\mathbf{r}) = \frac{1}{\sqrt{N}} \sum_{\mathbf{R}^{M,b}_{mn}} e^{\pm i\mathbf{K}\mathbf{R}^{M,b}_{mn}} Y_{2,\pm 2}(\mathbf{r} - \mathbf{R}^{M,b}_{mn}), \tag{45}$$

$$\Psi^b_{\pm\mathbf{K},c}(\mathbf{r}) = \frac{1}{\sqrt{N}} \sum_{\mathbf{R}^{M,b}_{mn}} e^{\pm i\mathbf{K}\mathbf{R}^{M,b}_{mn}} Y_{2,0}(\mathbf{r} - \mathbf{R}^{M,b}_{mn}). \tag{46}$$

We define the Bloch states of the top lattice in the same way

$$\Psi^t_{\pm\mathbf{K},v}(\mathbf{r}) = \frac{1}{\sqrt{N}} \sum_{\mathbf{R}^{M,t}_{mn}} e^{\pm i\mathbf{K}\mathbf{R}^{M,t}_{mn}} Y_{2,\pm 2}(\mathbf{r} - \mathbf{R}^{M,t}_{mn}), \tag{47}$$

$$\Psi^t_{\pm\mathbf{K},c}(\mathbf{r}) = \frac{1}{\sqrt{N}} \sum_{\mathbf{R}^{M,t}_{mn}} e^{\pm i\mathbf{K}\mathbf{R}^{M,t}_{mn}} Y_{2,0}(\mathbf{r} - \mathbf{R}^{M,t}_{mn}). \tag{48}$$

They have the corresponding transformation rules $\widehat{C}_3\Psi^t_{\pm\mathbf{K},v}(\mathbf{r}) = e^{\pm 2\pi i/3}\Psi^t_{\pm\mathbf{K},v}(\mathbf{r})$ and $\widehat{C}_3\Psi^t_{\pm\mathbf{K},c}(\mathbf{r}) = \Psi^t_{\pm\mathbf{K},c}(\mathbf{r})$. After rotation the basis states of the top layer get the phases which deviates from the corresponding phases of the basis states of the bottom layer. This difference is the result of the shift in y direction of the atoms of the top lattice with respect to the bottom one. Despite this difference, the optical transition rules are not changed for each separate layer of bilayer. Namely the top and bottom layers in $\pm\mathbf{K}$ points absorb the $\sigma^\pm$ circular polarized light, respectively. These optical properties can be understood as a consequence of time-reversal symmetry which couples $\mathbf{K}$ and $-\mathbf{K}$ points of bilayer. This feature demonstrates the significant difference in optical properties of 0°- and 60°-aligned S-TMD bilayers. The mirror symmetry transformation $\widehat{P}$ also couples $\pm\mathbf{K}$ points $\widehat{P}\Psi^\alpha_{\pm\mathbf{K},n}(\mathbf{r}) = [\Psi^\alpha_{\pm\mathbf{K},n}(\mathbf{r})]^* = \Psi^\alpha_{\mp\mathbf{K},n}(\mathbf{r})$, where $n = c, v$ and $\alpha = b, t$.

Like in previous section we focus on the states at the $\mathbf{K}$ point, take into account the spin degrees of freedom and introduce the basis states

$$|\Psi^b_c, s\rangle = \Psi^b_{\mathbf{K},c}(\mathbf{r})|s\rangle, \quad |\Psi^b_v, s\rangle = \Psi^b_{\mathbf{K},v}(\mathbf{r})|s\rangle, \tag{49}$$

$$|\Psi^t_c, s\rangle = \Psi^t_{\mathbf{K},c}(\mathbf{r})|s\rangle, \quad |\Psi^t_v, s\rangle = \Psi^t_{\mathbf{K},v}(\mathbf{r})|s\rangle. \tag{50}$$

We use the $\mathbf{kp}$ approach and calculate the matrix elements $\langle\Psi^\alpha_n, s|H(\mathbf{r})|\Psi^{\alpha'}_{n'}, s'\rangle$ of the one-particle Hamiltonian

$$H(\mathbf{r}) = \frac{\widehat{\mathbf{p}}^2}{2m_0} + U^b(\mathbf{r}) + U^t(\mathbf{r}) + \frac{\hbar}{4m_0^2c^2}[\nabla U^b(\mathbf{r}), \widehat{\mathbf{p}}]\boldsymbol{\sigma} + \frac{\hbar}{4m_0^2c^2}[\nabla U^t(\mathbf{r}), \widehat{\mathbf{p}}]\boldsymbol{\sigma} + \frac{\hbar}{m_0}\mathbf{k}\widehat{\mathbf{p}}. \tag{51}$$

All the terms in this Hamiltonian has the same meaning as in previous section. We also omit the $\mathbf{kp}$ term, since we are focused on the optical transitions exactly in $\pm\mathbf{K}$ points for clarity.

Let us consider the states of bottom layer. We present the Hamiltonian in the following form

$$H(\mathbf{r}) = H^b_0(\mathbf{r}) + H^t_{int}(\mathbf{r}), \tag{52}$$

where we introduced the Hamiltonian of the bottom monolayer

$$H^b_0(\mathbf{r}) = \frac{\widehat{\mathbf{p}}^2}{2m_0} + U^b(\mathbf{r}) + \frac{\hbar}{4m_0^2c^2}[\nabla U^b(\mathbf{r}), \widehat{\mathbf{p}}]\boldsymbol{\sigma}, \tag{53}$$

and the term which affects the motion of quasiparticles of the bottom layer by the crystal field of the top layer

$$H^t_{int}(\mathbf{r}) = U^t(\mathbf{r}) + \frac{\hbar}{4m_0^2c^2}[\nabla U^t(\mathbf{r}), \widehat{\mathbf{p}}]\boldsymbol{\sigma}. \tag{54}$$

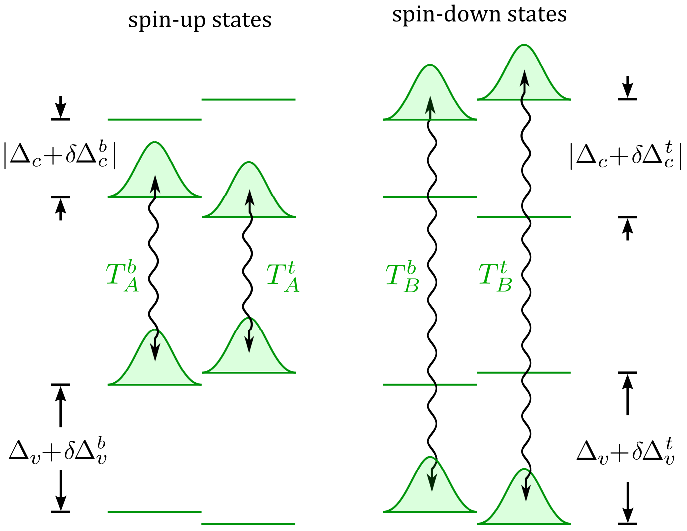

FIG. 2. The bands positions and optical transitions in the $+\mathbf{K}$ point of MoS_2 bilayer with $0°$-angle alignment. Left/right side represents the spin-up/spin-down states in the system. Green convexes represent the conduction and valence band states associated with optical transitions active in the σ^+ polarization. Wavy arrows indicates optical transitions T_A^b, T_A^t, T_B^b, T_B^t due to the intralayer A and B excitons in the botom (b) and top (t) layers. $|\Delta_v+\delta\Delta_v^b|$ ($|\Delta_v+\delta\Delta_v^t|$) and $|\Delta_c+\delta\Delta_c^b|$ ($|\Delta_c+\delta\Delta_c^t|$) denote the splitting in the conduction and valence bands of the bottom(top) layer, respectively.

The Hamiltonian $H_0^b(\mathbf{r})$ has the diagonal matrix elements

$$\langle\Psi_v^b, s|H_0^b(\mathbf{r})|\Psi_v^b, s\rangle = E_v + \sigma_s\Delta_v/2, \tag{55}$$

$$\langle\Psi_c^b, s|H_0^b(\mathbf{r})|\Psi_c^b, s\rangle = E_c + \sigma_s\Delta_c/2, \tag{56}$$

where all the parameters have the same meaning as in the previous section. The dominating contribution from of $H_{int}^t(\mathbf{r})$ has also diagonal matrix elements

$$\langle\Psi_v^b, s|H_{int}^t(\mathbf{r})|\Psi_v^b, s\rangle = \delta E_v^t + \sigma_s\delta\Delta_v^t/2, \tag{57}$$

$$\langle\Psi_c^b, s|H_{int}^t(\mathbf{r})|\Psi_c^b, s\rangle = \delta E_c^t + \sigma_s\delta\Delta_c^t/2, \tag{58}$$

This term also has non-zero matrix element between valence and conduction bands of opposite spins. As in previous case they give a negligibly small contribution to the energy of the bands, proportional to $\sim 1/(E_c - E_v)$. Therefore, we omit these matrix element from the current study.

The matrix elements between the states of the top layer can be calculated in the same way. Namely we present the Hamiltonian as

$$H(\mathbf{r}) = H_0^t(\mathbf{r}) + H_{int}^b(\mathbf{r}), \tag{59}$$

with the Hamiltonian of the top monolayer

$$H_0^t(\mathbf{r}) = \frac{\hat{\mathbf{p}}^2}{2m_0} + U^t(\mathbf{r}) + \frac{\hbar}{4m_0^2c^2}\left[\nabla U^t(\mathbf{r}), \hat{\mathbf{p}}\right]\boldsymbol{\sigma}, \tag{60}$$

and the term which affects the motion of quasiparticles of the top layer by the crystal field of the bottom one

$$H_{int}^b(\mathbf{r}) = U^b(\mathbf{r}) + \frac{\hbar}{4m_0^2c^2}\left[\nabla U^b(\mathbf{r}), \hat{\mathbf{p}}\right]\boldsymbol{\sigma}. \tag{61}$$

Again, only diagonal matrix elements of the $H_0^t(\mathbf{r})$ are nonzero

$$\langle\Psi_v^t, s|H_0^t(\mathbf{r})|\Psi_v^t, s\rangle = E_v + \sigma_s\Delta_v/2, \tag{62}$$

$$\langle\Psi_c^t, s|H_0^t(\mathbf{r})|\Psi_c^t, s\rangle = E_c + \sigma_s\Delta_c/2, \tag{63}$$

The dominating contribution from of $H^b_{int}(\mathbf{r})$ has also diagonal matrix elements

$$\langle \Psi^t_v, s|H^b_{int}(\mathbf{r})|\Psi^t_v, s\rangle = \delta E^b_v + \sigma_s \delta\Delta^b_v/2, \tag{64}$$

$$\langle \Psi^t_c, s|H^b_{int}(\mathbf{r})|\Psi^t_c, s\rangle = \delta E^b_c + \sigma_s \delta\Delta^b_c/2. \tag{65}$$

Note that due to the absence of the additional symmetry of the crystal (like inverse symmetry for 2H-stacked bilayers, or mirror symmetry for AA-stacked bilayer) we cannot find the relations between the parameters $\{\delta E^b_v, \delta\Delta^b_v, \delta E^b_c, \delta\Delta^b_c\}$ and $\{\delta E^t_v, \delta\Delta^t_v, \delta E^t_c, \delta\Delta^t_c\}$. Therefore, according to the symmetry analysis, we consider these parameters as independent ones.

Finally, we calculate the interlayer matrix elements $\langle \Psi^t_n, s|H(\mathbf{r})|\Psi^b_{n'}, s'\rangle$. Supposing the orthogonality of the states from the opposite layers and repeating the idea of the calculation from the previous section one gets

$$\langle \Psi^t_n, s|H(\mathbf{r})|\Psi^b_{n'}, s'\rangle = -\frac{1}{2m_0}\langle \Psi^t_n, s|\widehat{\mathbf{p}}^2|\Psi^b_{n'}, s'\rangle. \tag{66}$$

Since the $\widehat{\mathbf{p}}^2$ operator is a spin singlet, the matrix elements are diagonal in spin subspace $\langle \Psi^t_n, s|\widehat{\mathbf{p}}^2|\Psi^b_{n'}, s'\rangle = \delta_{ss'}\langle \Psi^t_n|\widehat{\mathbf{p}}^2|\Psi^b_{n'}\rangle$. Here we introduce the notation $|\Psi^\alpha_n\rangle = \Psi^\alpha_{\mathbf{K},n}(\mathbf{r})$. Using transformation properties of the basis states under C_3 rotation we get

$$\langle \Psi^t_v|\widehat{\mathbf{p}}^2|\Psi^b_c\rangle = \langle \Psi^t_v|\widehat{C}_3^{-1}\widehat{C}_3\widehat{\mathbf{p}}^2\widehat{C}_3^{-1}\widehat{C}_3|\Psi^b_c\rangle = e^{-4\pi i/3}\langle \Psi^t_v|\widehat{\mathbf{p}}^2|\Psi^b_c\rangle = 0, \tag{67}$$

$$\langle \Psi^t_c|\widehat{\mathbf{p}}^2|\Psi^b_c\rangle = \langle \Psi^t_c|\widehat{C}_3^{-1}\widehat{C}_3\widehat{\mathbf{p}}^2\widehat{C}_3^{-1}\widehat{C}_3|\Psi^b_c\rangle = e^{-2\pi i/3}\langle \Psi^t_c|\widehat{\mathbf{p}}^2|\Psi^b_c\rangle = 0, \tag{68}$$

$$\langle \Psi^t_v|\widehat{\mathbf{p}}^2|\Psi^b_v\rangle = \langle \Psi^t_v|\widehat{C}_3^{-1}\widehat{C}_3\widehat{\mathbf{p}}^2\widehat{C}_3^{-1}\widehat{C}_3|\Psi^b_v\rangle = e^{-2\pi i/3}\langle \Psi^t_v|\widehat{\mathbf{p}}^2|\Psi^b_v\rangle = 0, \tag{69}$$

$$\langle \Psi^t_c|\widehat{\mathbf{p}}^2|\Psi^b_v\rangle = \langle \Psi^t_c|\widehat{C}_3^{-1}\widehat{C}_3\widehat{\mathbf{p}}^2\widehat{C}_3^{-1}\widehat{C}_3|\Psi^b_v\rangle = \langle \Psi^t_c|\widehat{\mathbf{p}}^2|\Psi^b_v\rangle = \tau \neq 0. \tag{70}$$

The P symmetry of the crystal dictates that the parameter τ is a real number $\mathrm{Im}[\tau] = 0$. This parameter couples the valence band of the bottom layer with the conduction band of the top layer with the same spin state. It induces the negligibly small correction to the energies of the corresponding bands, proportional to $\sim 1/(E_c - E_v)$. Therefore, we omit such terms in our consideration.

Summarizing the aforementioned calculations we conclude that the states of the top and bottom layers are not mixed. Therefore, the considered bilayer has the same optical properties as a monolayer. Namely, this bilayer absorbs the $\sigma^\pm$ polarized light in the $\pm\mathbf{K}$ points that induces the intralayer dipole transitions A^b, B^b and A^t, B^t within the bottom and top layers, respectively. The energies of these excitons are

$$E_{A^b} = -\mathcal{E}_{A^b} + E_c + \delta E^b_c - E_v - \delta E^b_v + \frac{\Delta_c + \delta\Delta^b_c}{2} - \frac{\Delta_v + \delta\Delta^b_v}{2}, \tag{71}$$

$$E_{B^b} = -\mathcal{E}_{B^b} + E_c + \delta E^b_c - E_v - \delta E^b_v - \frac{\Delta_c + \delta\Delta^b_c}{2} + \frac{\Delta_v + \delta\Delta^b_v}{2}, \tag{72}$$

$$E_{A^t} = -\mathcal{E}_{A^t} + E_c + \delta E^t_c - E_v - \delta E^t_v + \frac{\Delta_c + \delta\Delta^t_c}{2} - \frac{\Delta_v + \delta\Delta^t_v}{2}, \tag{73}$$

$$E_{B^t} = -\mathcal{E}_{B^t} + E_c + \delta E^t_c - E_v - \delta E^t_v - \frac{\Delta_c + \delta\Delta^t_c}{2} + \frac{\Delta_v + \delta\Delta^t_v}{2}. \tag{74}$$

Here $\mathcal{E}_{A^b}, \mathcal{E}_{B^b}, \mathcal{E}_{A^t}, \mathcal{E}_{B^t}$ are the absolute values of the binding energies of the corresponding excitons. The band positions for the MoS_2 bilayer with $0°$-angle alignment is sketched in Fig. 2.

The energies of the top- and bottom-excitons of the same type (A or B) deviate from each other in general case. The symmetry analysis cannot provide the value of the splitting between A^b and A^t (B^b and B^t) exciton lines. However, as we do not observe any splittings of the A and B emission lines for $6°$ structure (see Fig. 3 in the main text), we suppose that the A^b-A^t and B^b-B^t splittings are quite small and probably cannot be recognized in the experiment. As a result, we assume that the experimentally obtained A-B energy distance equals $\Delta^{0°}_{A-B} = (E_{B^b} + E_{B^t})/2 - (E_{A^b} + E_{A^t})/2$. Similarly to the 2H-stacked MoS_2 bilayer, the splittings in conduction band are supposed to be much smaller than the splitting in valence band: $|\Delta_c + \delta\Delta^b_c| \ll \Delta_v + \delta\Delta^b_v$ and $|\Delta_c + \delta\Delta^t_c| \ll \Delta_v + \delta\Delta^t_v$, and the binding energies of A and B excitons are considered to be equal $\mathcal{E}_{A^b} = \mathcal{E}_{A^t} = \mathcal{E}_{B^b} = \mathcal{E}_{B^t}$. It implies that $\Delta^{0°}_{A-B} = (E_{B^b} + E_{B^t})/2 - (E_{A^b} + E_{A^t})/2 \approx \Delta_v + (\delta\Delta^b_v + \delta\Delta^t_v)/2$. Morevoer, if we assume that the $\delta\Delta^b_v \approx \delta\Delta^t_v \approx \delta\Delta_v$ (where $\delta\Delta_v$ is the corresponding parameter

calculated for 2H-stacked BL), then $\Delta^{0^\circ}_{A-B} \approx \Delta^{2H}_{v}$.

---

* magdalena.grzeszczyk@fuw.edu.pl
† maciej.molas@fuw.edu.pl

[1] G.-B. Liu, D. Xiao, Y. Yao, X. Xu, and W. Yao, Chem. Soc. Rev. **44**, 2643 (2015).
[2] M. R. Molas, K. Nogajewski, A. O. Slobodeniuk, J. Binder, M. Bartos, and M. Potemski, Nanoscale **9**, 13128 (2017).
[3] A. Arora, M. Koperski, A. Slobodeniuk, K. Nogajewski, R. Schmidt, R. Schneider, M. R. Molas, S. M. de Vasconcellos, R. Bratschitsch, and M. Potemski, 2D Materials **6**, 015010 (2018).
[4] A. O. Slobodeniuk, Ł. Bala, M. Koperski, M. R. Molas, P. Kossacki, K. Nogajewski, M. Bartos, K. Watanabe, T. Taniguchi, C. Faugeras, and M. Potemski, 2D Materials **6**, 025026 (2019).
[5] H. Ochoa and R. Roldán, Phys. Rev. B **87**, 245421 (2013).
[6] A. O. Slobodeniuk and D. M. Basko, 2D Materials **3**, 035009 (2016).
